# *Delphinium uncinatum* mediated biosynthesis of zinc oxide nanoparticles and *in-vitro* evaluation of their antioxidant, cytotoxic, antimicrobial, anti-diabetic, anti-inflammatory, and anti-aging activities

**DOI:** 10.1016/j.sjbs.2022.103485

**Published:** 2022-11-01

**Authors:** Hina Rehman, Waqar Ali, Nadir Zaman Khan, Muhammad Aasim, Tariq Khan, Ayaz Ali Khan

**Affiliations:** Department of Biotechnology, University of Malakand, Chakdara, Lower Dir 18800, Pakistan

**Keywords:** *Delphinium uncinatum*, ZnNPs, Antioxidant activities, Cytotoxic activities, Antimicrobial assays, Anti-diabetic activities, Anti-aging activities

## Abstract

Nanotechnology is perhaps the most widely explored scientific domain in the current era. With the advent of NPs, revolutionary changes have been observed in various scientific disciplines. Among the NPs, ZnO-NPs are the center of contemplation owing to their biocompatible nature. These nanoparticles have been prepared using a number of techniques; however, biological methods are among the most popular synthesis approaches. The current research therefore reports the phyto-fabrication of ZnO-NPs mediated by *Delphinium uncinatum* root extract. The resulting NPs were subjected to standard characterization methods such fourier transformed infrared spectroscopy, X-ray diffraction and transmission electron microscopy. The resulting NPs are exploited to their possible antioxidant, antimicrobial, antidiabetic, cytotoxic, anti-inflammatory and anti-ageing potency. FTIR confirmed the capping of ZnO-NPs by a variety of phytochemicals. ZnO-NPs average size was approximately 30 nm. ZnO-NPs exhibited substantial bio-potency and proved to be highly biocompatible even at higher concentrations. ZnO-NPs revealed strong antimicrobial potency for *Pseudomonas aeruginosa* proving to be the most susceptible strain showing inhibition of 16 ± 0.98. ZnO-NPs also showed dose dependent antidiabetic and cytotoxic potential. COX-1, COX-2, 15-LOX and sPLA2 were efficiently inhibited upon exposure to ZnO-NPs confirming the anti-inflammatory potential of ZnO-NPs. Similarly, ZnO-NPs also revealed considerable anti-aging potential. With such diverse biological potentials, ZnO-NPs can prove to be a potent weapon against a plethora of diseases; however, further study is necessary in order to discover the precise mechanism that is responsible for the biological potency of these NPs.

## 1 Introduction

Nanotechnology has the potential to revolutionize a range of scientific sectors. Nanomaterials offer a wide variety of uses owing to their morphology and size, and they have long been a hot topic in both fundamental and applied sciences. Nano-sized semiconductors are being attributed as a novel approach recently because their new characteristics have applicability in optoelectronics (Cao et al., 2011). There are two types of nanoparticle (NP) synthesis methods: chemical approaches and physical processes (Ghorbani et al., 2011). High cost, consumption of time, and more labor are some of the disadvantages of this procedure.

Furthermore, the inclusion of chemical agents for reduction and precipitation in the operations results in enormous amounts of secondary waste (Nagarajan and Arumugam Kuppusamy 2013). Because of such limitations, biosynthetic approaches utilizing bacteria, fungi, and algae, as well as some phyto components, like fruits, roots, stems, leaves, and seeds extracts, are quickly gaining attention as a simplified substitute and large-scale production technique for the synthesis of impurity-free metal oxide particles (Sri Sindhura et al., 2014). As a result, an alternative, safe, ecologically sound, and cost-effective method of NP synthesis is required (Narayanan et al., 2012).

Among different NPs, Zinc oxide NPs (ZnO-NPs) are flexible semiconductors with high luminescence and optical transparency in the UV–Visible (UV–Vis) range (Cao et al., 2011). As a result of their many uses in the communications, electronics, cosmetics, sensors, ecological protection, biological, and pharmaceutical sectors, ZnO-NPs have gathered the interest of many researchers (Cross, 2007, Wang et al., 2012, Dagdeviren et al., 2013). Because of their exceptional thermal and chemical stabilities, these NPs have become more essential in recent years (Nunes et al., 1999).

ZnO-NPs have a number of biological applications as well. ZnO-NPs have shown tremendous antibacterial and antifungal activities (Riaz et al., 2018). Owing to their GRAS (Generally recognized as safe) status as per FDA, the use of ZnO-NPs is preferred in various biological assays (Siddiquah et al., 2018). ZnO-NPs have also showed numerous applications in drug delivery and diagnostics (Sumaira et al., 2018). Research has also shown ZnO-NPs to have tremendous anti-cancerous potency (Zaka et al., 2021). ZnO-NPs are also known to influence seed germination, roots and shoot growth in a number of plants (Ali et al., 2022).

Several methods for synthesizing ZnO-NPs have been developed, like spray pyrolysis, sol–gel, zinc-alcohol reaction, microwave-assisted procedures, hydrothermal, ultrasonic conditions, chemical vapor deposition, and precipitation techniques (Omri et al., 2014). These preparations require a lot of energy and include poisonous and harmful substances, which might pose a biological risk.

On the other hand, biological approaches are more popular since they are frequently single-step, cost-effective, clean, and safe (Fakhari et al., 2019). Green synthesis methods are gaining popularity since they avoid the expensive costs for their costs as harsh chemicals and the harsh conditions required for reduction and stabilization (Mason et al., 2012). According to Raveendran et al., Biosynthetic procedures yield NPs with better-defined shapes and sizes than other physicochemical techniques (Raveendran et al., 2003). Natural compounds found in biological systems have an important and varied function in the NPs synthesis, and they serve as capping and stabilizing agents. The literature survey reveals that employing plants has significant benefits over other biosystems. Plants are readily accessible and convenient to use, and their NPs are more stable (Iravani 2011).

The *Delphinium uncinatum* Hook's & Thoms is an important herb with radical-shaped leaves and bluish-purple flowers. This plant, Known as Ranunculaceae, has a length of centimeters, about 15–90 found along the Himalayas across Pakistan and North-West India & Kashmir (Nasir 1991). *Delphinium uncinatum* is of medicinal value like its family member *Delphinium denudatum* Wall which has been considered and studied to be useful in a myriad of illnesses, such as analgesic, cardio, hepato- cellular protective, etc. (Suresh et al., 2014). However, unlike *Delphinium denudatum* Wall, its medicinal importance has not been fully explored, and most of its biological properties are not been reported so far. So the current study is considered and executed to study the ZnO-NPs biosynthesis by using the *Delphinium uncinatum-based* root aqueous extract by Hooks and Thomas, along with characterization and some biological activities.

## 2 Material and methods

### 2.1 Collection of plant material and preparation of aqueous extract

*Delphinium uncinatum* Hook's & Thoms was collected from KP (Swat) and was identified for taxonomic classification at the Department of Botany, University of Swat. The plant roots were collected and washed thoroughly using tap water before subjecting to shade-drying. After drying, the roots were finely powdered using an electrical grinder. The powdered material of roots (10 g) was mixed with Distilled water (1L). The mixture was heated to 90 °C for 2 to 3 h. Finally, the *Delphinium uncinatum* root extract (DURA) was filtered using two screens, i.e., nylon cloth and Whatman filter paper. The extract was kept at 4 °C before usage.

### 2.2 **Green** synthesis **of ZnO-NPs**

The synthesis of ZnO-NPs was achieved through the co-precipitations approach with minor modifications (Dadalioǧlu and Evrendilek 2004). To summarize, DURA (150 mL) were combined with a solution containing of zinc acetate dihydrate (150 mL). The mixture was then placed on a magnetic stirrer and left there for two hours while the pH was maintained constant at 12. The mixture was kneaded until a yellow-colored solid was formed. The yellow residue was then purified by washing it thrice with distilled water, followed by centrifugation. The final pellet was dried at 60 °C to produce a white powder. Thermal decomposition was used to eliminate contaminants from biosynthesized ZnO-NPs after calcining at 400 °C for three hours.

### 2.3 Characterization **of ZnO-NPs**

Fourier transform infrared spectroscopy (FTIR) was used to detect different functional groups that played their role as reducing, capping, and stabilizing agents during the biologically synthesized of ZnO-NPs and their spectral properties. FTIR was used inside spectral range of 400 to 4000 cm^−1^. Transmission electron microscopy (TEM) is normally utilized to determine the size, form, and shape of the biologically synthesized ZnO-NPs. This was achieved by means of a Philips EM201C device operating at 80 kV. For TEM measurements, the samples were prepared in a high dispersion of 2-propanol and then placed on a copper grid coated with carbon.

X-ray diffraction (XRD) was used so that a crystal structure for the ZnO-NPs could be predicted. Cathode rays produce X-rays and are emitted by the XRD instrument, which passes materials through. From 0° to 80° in the 2θ zone, the structure of ZnO- NPs was investigated and analyzed. The Debye-Sherrer equation was used in order to determine the crystalline size of the ZnO- NPs. using the equation that is below:

K=dλ/αCosθ

Where the different symbols are as,

d = Shape Factor (0.94),

λ = X-rays wavelength (1.5421 Å),

**α** = full width at half maximum in radians, and.

θ = Bragg’s Angle.

### 2.4 Phytochemical **analysis of ZnO-NPs**

#### 2.4.1 Total phenolic and flavonoid content

The Folin–Ciocalteu technique, with a little modification, was used to perform the analysis for the Total Phenolic Content (Antony et al., 2013). The NPs samples (4 mg/mL) were moved into 96 well plates, and 90 µL of Folin–Ciocalteu reagent was added to each well and reaction mixture was nurtured for five minutes before sodium carbonate was add alsoed to the plate. The absorbance of the reaction mixture after one hour incubation was measured at 630 nm using a microplate reader (Biotek USA, microplate reader Elx 800). The sample of gallic acid that was utilized was considered as the standard. The results obtained were presented as per milligrams of the gallic acid equivalents (GAE/mg).

The Total flavonoid content was also calculated using the aluminum chloride method (AlCl_3_) (Paulkumar et al., 2014). Each well of the plate containing 10 % AlCl_3_ solution, purified water, and potassium acetate was filled with 20 µL of NP sample (4 mg/mL). After 30 min of incubation at room temperature, the plate was analyzed. The microplate reader recorded the sample absorbance at 630 nm. Two different substances, quercetin and DMSO, served as positive and negative controls, respectively. Three replicate reactions were used to calculate the quercetin equivalent per milligrams (QE/mg) in the final extracts.

### 2.5 Antioxidant **activities**

#### 2.5.1 DPPH-free radical scavenging

We used the 2,2- Diphenyl-1-picrylhydrazyl (DPPH) assay to determine the Free radical scavenging capacity for biosynthesized ZnO-NPs at low concentrations i.e. 10 µg/mL up to 200 µg/mL (Ali et al., 2017a, Ali et al., 2017b). For the controls, both positive and negative, ascorbic acid and DMSO were used. Reagent solution (180 µL) and ZnO-NPs samples (20 µL) were mixed separately to make a total reaction mixture of 200 µL. After incubating for one hour, the absorbance at 517 nm was recorded to determine the % of scavenging ability of the examined materials by the following formula.

% Scavenging = 1 − ABS / ABC × 100, where.

“ABS” and “ABC” represent the absorbance of different samples and control.

#### 2.5.2 **Total** antioxidant **capacity**

The phosphate molybdenum method was used to calculate ZnO-NPs' total antioxidant capability (Jafri et al., 2017). To generate 200 µL of reaction, we introduced 180 µL of the reagent combination (0.6 M H_2_SO_4_, 4 mM (NH_4_)_6_Mo_7_O_24_, 4H_2_O, 28 mM NaH_2_PO_4_) to 20 µL of test samples. After that, the plates went into a 95 °C incubator for 90 min. The absorbance at 695 nm was used to convert the results into micrograms of ascorbic acid equivalents per milligram of sample, abbreviated as AAE/mg.

#### 2.5.3 Total **reducing power**

ZnO-NPs produced using potassium-ferricyanide (K_3_Fe (CN)_6_) based technique was tested for their total reducing power (Javed et al., 2016). A 50 µL potassium phosphate buffer (PBS) with 40 µL of test samples was allowed to incubate for 20 min at 50 °C. Centrifugation of the reaction mixtures was performed for 10 min at 3000 rpm following the addition of trichloroacetic acid (10 %, 50 µL). In a 96-well plate, 166.6 µL of supernatant and 33.3 µL of FeCl_3_ were combined with the supernatant (0.1 %). Expressed as AAE/mg, the proposed research established that ascorbic acid and DMSO may be used as positive and negative controls, respectively. At the end of the procedure, the absorbance of the sample was calculated at 630 nm.

#### 2.5.4 Antimicrobial activities

Agar well diffusion was used against *Klebsiella pneumoniae*, *Bacillus subtilis*, *Pseudomonas aeruginosa*, and *Staphylococcus epidermidis* to test the antibacterial properties of ZnO-NPs (da Silva et al., 2019). To create a uniform bacterial lawn, a sterile cotton bud was used to disseminate microbial inoculum prepared the day before to an optical density (OD) of 0.5 throughout the whole surface of the agar plate. Following this, approximately 6 mm holes were punched in the prepared plate through a sterile hole borer, and a 20 µL volume of samples were added to each well. Various concentrations (1 mg, 2 mg, 4 mg, 5 mg, and 10 mg) of the biosynthesized ZnO-NPs were introduced into the specifically labeled well with positive (penicillin), and negative (DMSO) controls. The prepared plates were then placed in a bacterial incubator for 24 h and 48 h, respectively. Green synthesis ZnO-NPs as antimicrobial agents were diffused into the growth medium and produced growth inhibition zones that were measured using a vernier caliper.

### 2.6 Anti-diabetic activity

To check the ZnO-NPs potential as antidiabetic agent, the alpha-amylase inhibition assay following the procedure described in (Zohra et al., 2019) with a few changes were employed. To prepare the reaction mixture, 10 µL test samples (10 µg/mL to 200 µg/mL) concentration of ZnO-NPs), 15 µL PBS (pH 6.8), 40 µL starch solution and 25 µL alpha-amylase enzyme (0.14 U/ml) was added to a 96-well plate. 20 µL of 1 M HCl was added to each well, followed by 90 µL of iodine reagent to stop the reaction. Acar As a positive control for acarbose (250 M), whereas DMSO was used as a negative control. The following formula was presented to determine the level of alpha-amylase inhibition in a proposed formula.

%α-amylaseinhibition=(As-An)/(Ab-An)×100

Where,

*As* = Absorbance of the sample, *An* = Absorbance of negative control, and *Ab* = Absorbance of the blank well.

### 2.7 Cytotoxic activities

#### 2.7.1 Biocompatibility studies by hemolytic assay

Tests of ZnO-NPs’ human RBC hemolytic compatibility, or biocompatibility, were carried out using fresh blood (Abbasi et al., 2019). Fresh blood was drawn from three healthy students (one female and two males) who gave their consent and had no history of sickness. A fresh blood sample was centrifuged at 14,000 revolutions per minute for five minutes to obtain RBC pellets suspended in PBS (pH 7.2) in order to collect erythrocytes, a suspension of them must first be prepared. After incubation of the erythrocyte suspension and ZnO-NPs solution at 35 °C for 1 h, the mixture was centrifuged at 10,000 rpm for 10 min. The absorbance of the supernatant was evaluated at 530 nm in order to calculate the hemoglobin release rate. The positive and negative controls were, respectively, DMSO and Triton X-100.

The following equation was used to determine the percent Hemolysis.

$$\%Hemolysis=\frac{AB_{S}-AB_{NC}}{AB_{PC}-AB_{NC}}\times 100$$

Where,

“ABs” represents the absorbance of supernatant_,_

“AB _NC_” denotes negative control, and.

“AB_PC_” represents the absorbance of positive control.

#### 2.7.2 **Cell** viability **assay (XTT assay)**

ZnO-NPs are shown to have a detrimental effect on cell viability. Hence an XTT Cell viability assay was used to measure this effect (Choudhery et al., 2012). NIH 3 T3 murine fibroblast cell viability assay kit was used to test cytotoxicity for this objective (Roche, Switzerland). The fibroblast cells culture in DMEM_LG media supplement and 20 % fetal bovine serum were harvested using 1X Trypsin + EDTA and seeded 3,000 cells in each well of 96-well plates. For 24 h, the cell-seeded plate was kept at 37 °C in a 5 % CO_2_ incubator. All wells were incubated with serum-free media for 24 h treated after overnight incubation with varying concentrations of ZnO-NPs (25, 50, 75, and 100 µg/ml respectively) before being placed again in the CO_2_ incubator at 37 °C. Wells were washed twice with 1X PBS, and fresh XTT reagent and DMEM were added to each well according to directions provided in the kit after 24 h of treatment with NPs. For four hours, the plate was incubated in a CO_2_ incubator at 37 °C. Absorbance with a microplate reader was checked at 450 nm and 630 nm wavelengths. Three separate experiments were conducted.

### 2.8 **Anti-**inflammatory **activities**

#### 2.8.1 COX-1 and COX-2 inhibitory activities

ZnO-NPs' ability to inhibit COX-1 and COX-2 was evaluated using the COX-1 (Ovine kit 701050) and COX-2 assay kits in accordance with the protocol of (Nagajyothi et al., 2015). 10 mM ibuprofen was applied as a positive control, while 1.1 mM arachidonic acid was used as a substrate. The capacity of ZnO-NPs to prevent COXs peroxidase activity was evaluated using the instructions provided with the kit. By measuring the absorbance at 590 nm, the Synergy II reader (BioTek Instruments, USA) was utilized to colorimetrically identify N, N, N/, N/-tetramethyl-p-phenylenediamine in 96 well plates.

#### 2.8.2 **Inhibitory** activity **against 15-LOX**

According to the technique, ZnO-NPs potential to constrain the 15-LOX enzyme was tested by (Nagajyothi et al., 2015). Arachidonic acid (10 M) served as the substrate to ensure that the reaction proceeded as expected, and 100 M NDGA served as the positive control. To measure hydroperoxide concentrations produced by the lipo oxygenation reaction, we used a 15-lipoxygenase standard enzyme, ZnO-NPs, and the assay buffer included in the kit. The 10 L substrate (arachidonic acid) was poured into the wells and, at room temperature, incubated for 5 min with the 15-lipooxygenase enzyme. To stop the enzyme's catalysis, 10 L of chromogen was added to the plate, and it was let to sit at room temperature for an additional 5 min. An instantaneous measurement of the absorbance at 940 nm was made using the Synergy II reader**.**

#### 2.8.3 **Inhibitory** activity **against secretory phospholipase A2 (sPLA2)**

The Cayman Chem. USA test kit (10004883) was used to assess the ZnO-NPs produced during biosynthesis's inhibitory effects on the sPLA2 enzyme (Nagajyothi et al., 2015). Thyotheramide-PC (100 mM) served as both the reaction's substrate and a positive control. We used Synergy II and DTNB to detect the free thiols produced by the cleavage of diheptanoyl thio-PC ester (5–50-dithio-bis-butyl).

### 2.9 Anti-aging activities

#### 2.9.1 Estimation **of anti-tyrosinase activity**

L 3, 4-dihydroxyphenylalanine (l-DOPA, 5 mM) was used as a substrate and kojic acid (1.4 mg/mL) as a standard inhibitor to test the anti-tyrosinase activity of ZnO-NPs (Mostafa et al., 2019). l-DOPA was added after the mushroom tyrosinase (0.2 mg/mL) was incubated with ZnO-NPs (10 mL) and kojic acid for 15 min at 37 °C. A microplate reader was utilized to detect the dopachrome that had developed at a wavelength of 475 nm. ZnO-NPs inhibited the activity of the tyrosine enzyme, which was measured in percent.

#### 2.9.2 Anti-AGE formation activity

Vesperlysine and Pentosidine AGE generation inhibition potential was evaluated following an established technique (Mostafa et al., 2019). The BSA solution was prepared by adding glucose (0.5 M solution) and PBS solution (0.1 M solution) with 0.02 percent sodium azide (w/v). ZnO-NPs were added to the solution at 20 mg/mL. After being created, the reaction mixture was allowed to sit at 37 °C in the dark for five days. The fluorescence was measured using a VersaFluor fluorometer at an emanation wavelength of 410 nm and an excitation range of 330 nm.

#### 2.9.3 Hyaluronidase assay

A combination of hyaluronic acid and hyaluronidase (1.5 units; Sigma Aldrich) was mixed to create a substrate solution to test ZnO-NPs for their inhibitory ability against hyaluronidase. Undigested hyaluronic acid was precipitated with a BSA-containing acid albumin solution (0.1 percent w/v). A microplate reader measured the absorbance at 600 nm. It was measured in terms of the percentage of inhibition of NPs in comparison to the control**.**

#### 2.9.4 Estimation **of anti-elastase activity**

ZnO-NPs were incubated with porcine pancreatic elastase in a 96-well microtiter plate for 20 min at 25 °C to determine their anti-elastase activity. One milliliter of *N*-Methyl Succinic acid (AAAVPN, 1 mM) was then added to the mixture and incubated for additional 40 min following the first incubation period of twenty minutes. At 410 nm, a microplate reader measured the absorbance of the reaction mixture, which corresponds to the production of p-nitroaniline by the substrate. This was performed twice in triplicates.

#### 2.9.5 Estimation **of anti-collagenase activity**

Collagenase type 1 (1 mg/ml), ZnO-NPs, and buffer (pH 7.4) were incubated at 37 °C for 20 min in a 96-well microtiter plate to check the anti-collagenase activity of ZnO-NPs. Using FALGPA as a substrate, the plate was incubated at 37 °C for 1 h after 20 min with 100 L of 2-furyl]-acryloyl]-Leu-Gly-Pro-Ala (FALGPA). Afterward, 200 L of ninhydrin (200 mM, pH 5) and 200 L of isopropanol (200 mL) were added to the precooled reaction mixture and heated. anti-collagenase activity was expressed in percent inhibition at 540 nm at the end of the experiment.

## 3 Results

### 3.1 Biosynthesis of ZnO-NPs

In the present study, DURA was used to stabilize and reduce ZnO-NPs. The changes in the color of the reaction mixture upon visual observation serves as an indicator of ZnO-NPs synthesis once the zinc salt and DURA are mixed. The appearance of a white precipitate as an end product in the reaction mixture was the preliminary indication of the reduction of zinc and the successful synthesis of ZnO-NP. The main bioactive constituents present in *Delphinium uncinatum* include flavonoids, alkaloids (staphisagrine, condelphine, delphocurarine, talatizidine, delphine, isotalatizidine, hetisinone, denudatin, panicutine, delnudine, vilmorrianone, delnuline, vilmorri anonymous) terpenoids (14β-acetyl condelphine, 1βhydroxy, jadwarine-A, and jadwarine-B) and reducing sugars (Aleem et al., 2020). It has been reported previously that the aldehyde groups impart a significant role in reducing ZnO salt to form NPs (Nath and Banerjee, 2013, Singh et al., 2019). Thus, we can say that the DURA might have acted as a potential dropping and capping agent during the making of ZnO-NPs.. After washing and drying operations, white fine powder of ZnO-NPs was produced and put in an airtight jar before being used to characterize and evaluate biological potential.

### 3.2 Physicochemical **and Morphological characterization**

**Fig. 1:**
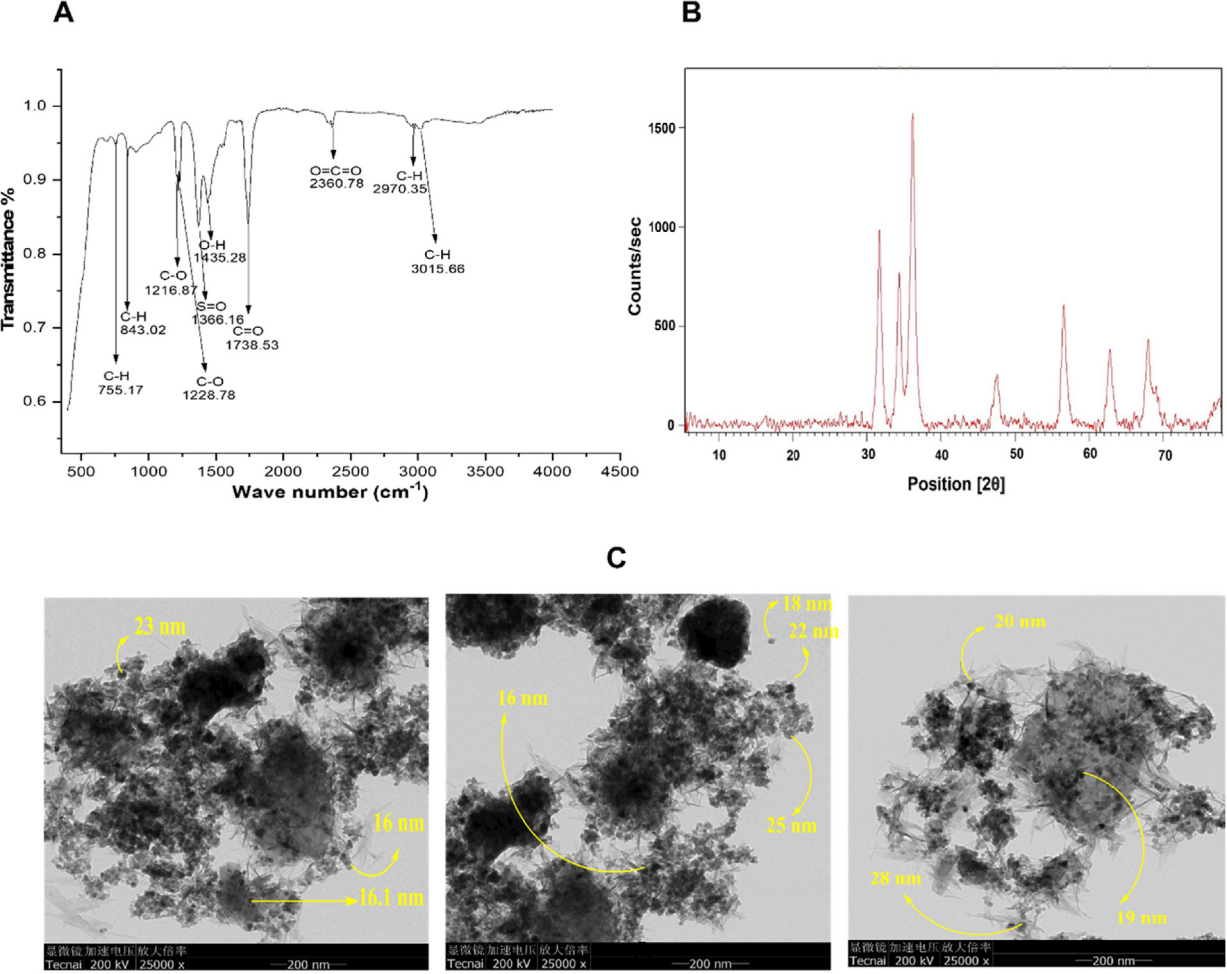
Physicochemical and Morphological Characterization of the ZnO-NPs. Figure (A) shows the FTIR spectrum of ZnO-NPs, (B) shows the XRD spectrum of ZnO-NPs, and (C) exhibits representative TEM images of ZnO-NPs.

DURA mediated ZnO-NPs was subjected to FTIR analysis to detect functional groups that might be the source of production for ZnO-NPs. At the region of 500–4500 cm^−1^, the ZnO-NPs FTIR spectrum was obtained, as shown in Fig. 1. (A). The peaks are seen at 3015 cm^−1^, 2970 cm^−1^, 845 cm^−1^, and 755 cm^−1^ pointing to C—H stretching vibrational amplitude vibration. C=O stretching can be seen in the band at 1738 cm^-1^and 1368 cm^−1^. CO_2_ stretching vibrations were responsible for the large peak at 2380 cm^−1^. O—H stretching vibration may have a role in the absorption peak at 1435 cm^−1^. The ZnO stretching vibration peak at 755 cm^−1^ confirms the production of ZnO-NPs (Huang et al., 2007, Arakha et al., 2015, Henry et al., 2015, Paithankar et al., 2015).

As shown in Fig. 1, numerous strong XRD peaks were found at various 2θ positions, including 68.03°, 62.82°, 56.51°, 47.53°, and 36.11°, which indicate the presence of crystallized ZnO-NPs in the biosynthesized (Fig. 1). (B). These findings support the conventional hexagonal wurtzite structure found in JCPDS file no:361451 and are consistent with those of (Janaki et al., 2015, Vijayakumar et al., 2016, Matinise et al., 2017), who demonstrated nearly identical 2θ values for ZnO-NPs.

According to the Debye Scherrer equation and XRD measurements, the usual size of biosynthesized ZnO-NPs was 30 nm. Fig. 1 illustrates the TEM analysis of biosynthesized ZnO-NPs (C).

Using TEM, it was discovered that the ZnO-NP particles had a size range of 16–28 nm, comparable to the average ZnO-NP particle size of 30 nm as determined by XRD.

### 3.3 Antioxidant **activities**

In general, antioxidants are substances capable of scavenging reactive species by halting oxidation reactions to prevent the deterioration of cells by ROS overproduction. Many natural components present in plant extracts can act as antioxidants. These natural antioxidant compounds are in enormous demand owing to their effective potential in controlling ROS-mediated pathogenesis of various degenerative diseases, including carcinogenesis and cardiovascular diseases (Nagajyothi et al., 2014, Gupta et al., 2019). DPPH, TAC, and TRP are different tests to verify the antioxidant capacity of biosynthesized ZnO-NPs. This method is being used to check the antioxidants' capability to scavenge in various foods, including juices, vegetables, extracts, and biosynthesized nanomaterials. It is simple, inexpensive, and highly sensitive (Sowndhararajan et al., 2013).

As shown in Table 1, the antioxidant capability of biosynthesized ZnO-NP was dramatically increased in the experiments using five different concentrations (i.e., 10, 50, 100, and 200 µg/mL). Mo (VI) is reduced to Mo (V) by ZnO-NPs, and a greenish Mo (V)-phosphate complex is formed, which has a maximum absorption wavelength of 695 nm. The total antioxidant capacity (TAC) of ZnO-NPs is resolute by this process (Saleem et al., 2014). Moderate (75.61 ± 1.06) ZnO-NP TAC activity was identified at concentration fixed at 200 µg/mL, which was further lowered to 25.27 ± 1.53 at 10 µg/mL dosages to find a maximum of 25.27 ± 1.53. A power reduction test demonstrated the antioxidant activity of ZnO-NPs (TRP). Redox-capable agents can absorb and neutralize free radicals by transferring ions from ferrous to ferric ions (Fe + 3 to Fe + 2). These reducing agents can donate hydrogen and reduce free radicals by dissolving free radical bonds. The potency of this reduction is directly correlated with the sample's antioxidant capacity (Moreira et al., 2008). Fe^+3^ can be reduced to Fe^+2^ using ZnO-NPs with an efficient, reducing potential (Saleem et al., 2014). When ZnO-NP was tested at 200 µg/mL, TRP analysis showed the highest antioxidant potential (110.34 ± 1.14 µg/mg AAE/mg) and the lowest activity (20.72 ± 1.74 µg AAE/mg) to be at a concentration of 10 µg/mL. A measure of antioxidant activity known as ascorbic acid equivalents describes all of these substances. Harbouring the quenching ability of DPPH free radicals to scavenge antioxidants, spectrophotometric DPPH scavenging activity is quantified (Zia-Ul-Haq et al., 2012). DPPH assay results showed that ZnO-NPs at 200 µg/mL had the highest radical scavenging activity (25.12 ± 1.48 µg AAE/mg) and the lowest radical scavenging activity (7.4 ± 1.42) at 10 µg/mL. However, considering the overall results of all antioxidant activities (DPPH, TRP, and TAC), the TRP assay (110.34 ± 1.14) at the 200 µg/mL measured concentration of ZnO-NP had the maximum antioxidant potential. In addition, as ZnO-NP concentrations rose, the reducing potential of the ZnO-NPs was found to increase with DPPH, TRP, and TAC activities.

**Table 1:** Antioxidant Potential of biosynthesized ZnO-NPs.

**Conc. (µg/mL)**	TRP (µg AAE/mg)	TAC (µg AAE/mg)	DPPH (% FRSA)
**200**	110.34 ± 1.14^***^	75.61 ± 1.06^***^	25.12 ± 1.48^**^
**100**	89.2 ± 1.45^**^	51.3 ± 0.78^**^	20.10 ± 1.26^**^
**50**	50.10 ± 0.94^***^	48.9 ± 0.82^**^	15.2 ± 1.94^**^
**10**	20.72 ± 1.74^**^	25.27 ± 1.53*	7.4 ± 1.42**

∗∗∗A highly significant, ∗∗moderately significant, and ∗∗slightly significant difference (P < 0.05) linked to the regulator was considered as examined by one-way ANOVA. Values are presented as mean ± SD of triplicate. Since aqueous root extracts of *Delphinium uncinatum* were utilized for the biosynthesis of ZnO-NPs, it can be considered that the phenolic compounds from the extract involved in the reduction and capping of ZnO-NPs may have quenched the reactive oxygen species as illustrated by findings of antioxidant activities.

### 3.4 Antibacterial **activities**

**Fig. 2:**
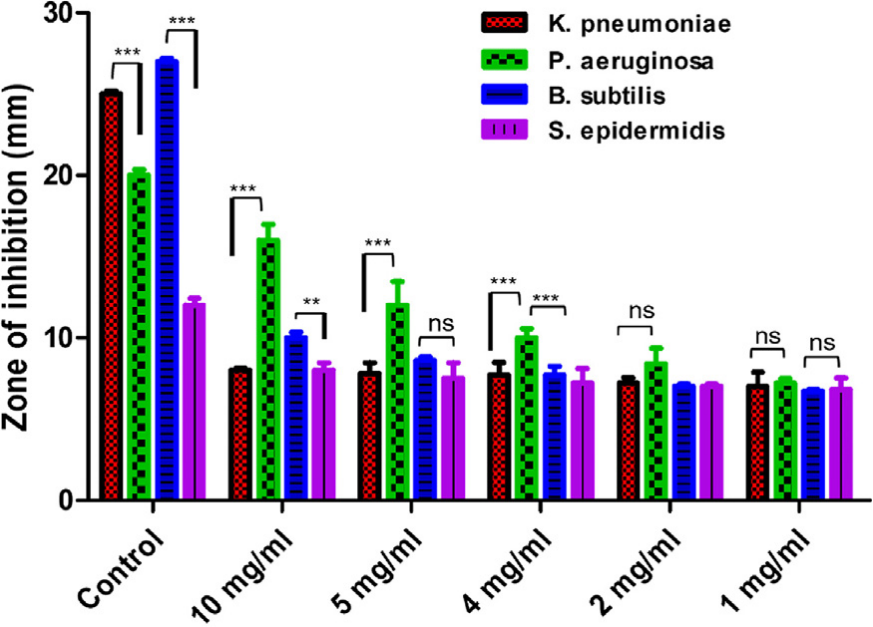
Graphical representation of the antibacterial activity of biosynthesized ZnO-NPs against four different bacterial strains.

**Fig. 3:**
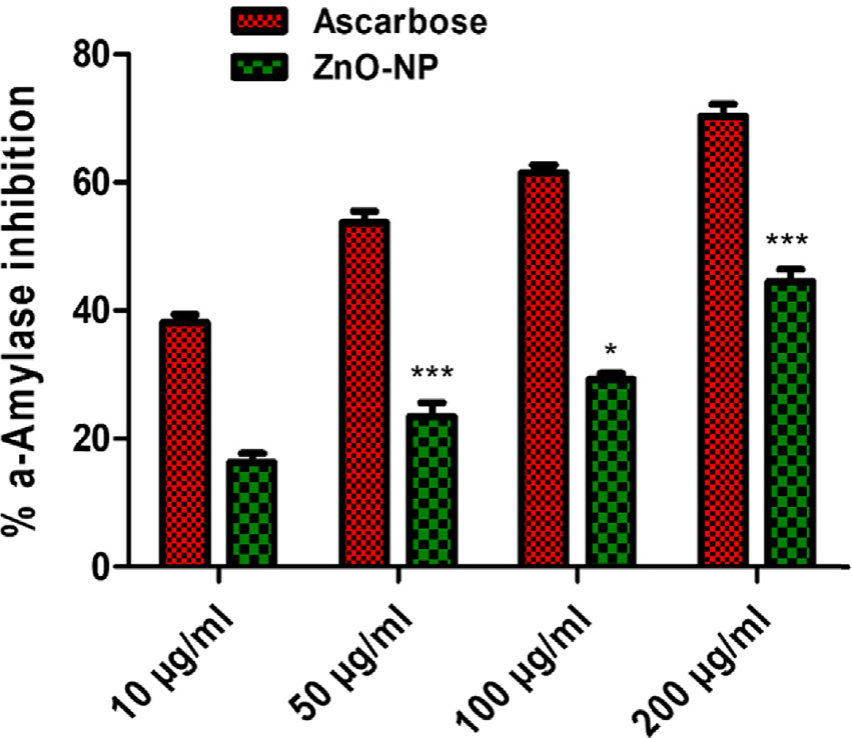
Graphical representation of the antidiabetic potential of ZnO-NPs via Amylase inhibition assay.

Two gram-negative bacteria and four major pathogenic bacterial strains were used to test the antibacterial properties of biosynthesized ZnO-NPs., i.e., *Klebsiella pneumoniae* and *Pseudomonas aeruginosa*, and two gram-positive strains, i.e., *Staphylococcus epidermidis* and *Bacillus subtilis****.*** Cefixime was used as a positive control against all tested bacterial strains. ZnO-NPs showed maximum inhibition against *Pseudomonas aeruginosa* (16 ± 0.98) compared to the cefixime control (20 ± 0.34). *Staphylococcus epidermidis* also exhibited a good inhibitory effect of (8 ± 0.45) compared to the respective control (cefixime) (12 ± 0.42). Overall, ZnO-NPs demonstrated efficient inhibition against all the tested concentrations of bacterial strains, as exhibited in Fig. 2; however, against *Pseudomonas aeruginosa,* the dose-dependent inhibitory effect of ZnO-NPs was achieved compared to other bacterial strains, and considerably higher inhibition was exhibited at 10 mg/ml and 5 mg/ml concentrations. The antimicrobial effect of biosynthesized ZnO-NPs is not clear. However, overproduction of ROS and RNS (reactive nitrogen species) are assumed to hamper the bacterial defense system by disturbing the mitochondrial membrane, cell membrane, and underlying organelles. ZnO-NPs showed a biocidal effect via ROS and RNS production, which tend to distort bacterial species' cell walls and cell membranes, leading to their death (Abinaya et al., 2017).Fig. 3.

### 3.5 Antidiabetic **activity**

Diabetic Mellitus (DM) is marked by an elevated blood glucose level (hyperglycemia) and is caused by a deficiency in the body's ability, to synthesize or respond to insulin (Nair et al., 2013). There are reports of 425 million adults having diabetes in the year 2017 alone, and that number is predicted to rise to 629 million by 2045, as estimated by the International Diabetes Federation (Aynalem and Zeleke, 2018). Postprandial hyperglycemia is lowered by suppressing enzymes in the digestive system that break down carbs, and alpha-amylase is one of those enzymes specifically targeted by medications (Nair et al., 2013). *In-vitro* tests for alpha-amylase inhibition used a range of ZnO-NP doses (10 µg/mL to 200 µg/mL). The data shows that ZnO-NPs had a dose-dependent inhibitory effect on alpha-amylase activity. The maximum inhibitory impact of ZnO-NPs against alpha amylase was observed at a concentration of 200 µg/mL, while the least inhibitory effect for ZnO-NPs was found at a concentration of 10 µg/mL (Fig. 3). Consequently, the modest antidiabetic activity of DURA mediated ZnO-NPs may be due to bioactive chemicals in the root extract that inhibits enzymes associated with diabetes. The experiment was carried out twice in three separate experiments.

### 3.6 Cytotoxicity **evaluation of ZnO-NPs**

#### 3.6.1 Biocompatibility with RBCs

**Fig. 4:**
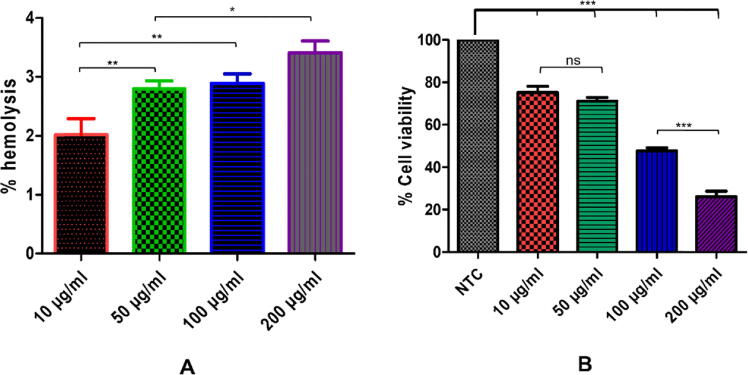
Cytotoxicity evaluation of biosynthesized ZnO-NPs: (A) shows % hemolysis of synthesized ZnO-NPs, and (B) shows cytotoxicity studies of ZnO-NPs on NIH 3T3 cells viability.

Biocompatibility assessment of NPs is an important factor in evaluating their potential biomedical applications. The hemolysis assay is based on the ability of synthesized NPs to induce lysis of red blood cells and estimate the amount of released hemoglobin from the RBCs after lysis. The hemoglobin count is observed via a spectrophotometer in the suspension media. These findings suggest that ZnO-NPs exhibited a hemolytic potential of 2 % at 10 µg/mL tested concentration of ZnO-NPs, while 200 μg/mL concentration showed increased hemolysis of 3.42 % respectively (Fig. 4A). It has been reported that material is considered hemolytic if it exhibits hemolysis of ≥ 5 %, and materials with 2–5 % hemolytic ability can be termed as slightly hemolytic while ≤ 2 % are considered non-hemolytic (Aula et al., 2014).

#### 3.6.2 XTT cell viability assay

Fibroblasts cells (NIH3T3) determined the cytotoxic impact of biogenic ZnO-NPs. After conducting tests using various concentrations of the ZnO-NPs, the results showed that the cytotoxicity of the NPs was dose-dependent. This suggest that the NPs showed less toxicity at lower concentrations but showed more toxic nature at higher concentrations. The results from the XTT assay showed a cell viability of 100.0 ± 0.012 % in the control group, compared to 75.23 ± 1.866 % in the 25 µg/ml concentration, 71.10 ± 1.784 % in the 50 µg/ml concentration, 55.63 ± 1.468 % in the 75 µg/ml concentration, and 52.13 ± 1.64 % in the 100 µg/ml concentration, respectively (Fig. 4B). When compared to the control and the other concentrations of NPs that were tested (50, 75, and100 µg/ml), the concentration of NPs that was tested at 25 µg/ml showed the least amount of toxicity to the cells. Cell viability was tested at quantities of 25 and 50 µg/ml, however the results showed no significant difference between the two. Both the 75 µg/ml and the 100 µg/ml concentrations of NPs exhibited the same general pattern of behavior. When compared to other concentrations, the 100 µg/ml concentration demonstrated the lowest level of cell viability and the highest level of toxicity. This is because it decreased the total vitality of the cells to 50 %. The conclusion that can be drawn from these findings is that concentrations of 100 µg/ml or above may seem to be toxic to the cells. Previous research also found that ZnO-NPs had better cell viability at lower concentrations. This was shown to be the case at lower concentrations. Because both time and concentration have an immediate effect on the viability of cells, it is dependent on both (Saranya et al., 2017).

### 3.7 *In-vitro* anti-Inflammatory potential

**Fig. 5:**
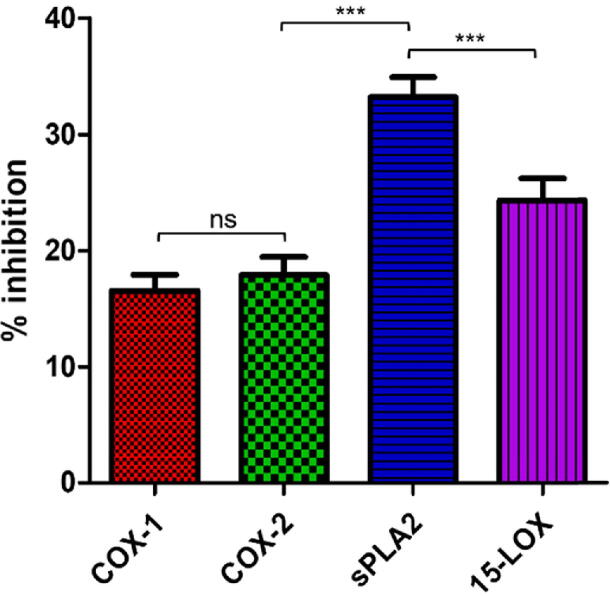
Anti-inflammatory potential (% inhibition) of biosynthesized ZnO-NPs.

Inflammation is a mechanized response that defends the body against various harmful agents, bacteria, irritants, adverse stimuli, and damaged cells. Various in-vitro and in-vivo anti-inflammatory activities have been reported for various metallic NPs and many secondary metabolic compounds. Biosynthesis of ZnO-NPs may involve flavonoid capping such as vitexin, isovitexin, orientin, and isoorientin. Flavonoid compounds possess efficient anti-inflammatory potential, including cyclooxygenase inhibition activity against COX-1, COX-2, and eicosanoids, developing enzymes such as lipoxygenases and phospholipase A2, reducing the level of prostanoids and leukotrienes which are involved in inflammation (Rathee et al., 2009). Certain current and emerging therapies can cope with inflammatory disorders by reducing the symptoms of inflammatory conditions to a greater extent (Gendelman et al., 2015). It has been reported that metallic NPs have the unique capability to infiltrate the microbial cell membrane. Hence this problem can be resolved by escalating penetration of a specific drug in case of microbial infection. In recent science, NPs are specifically synthesized to circumvent and manage inflammatory conditions by incorporating anti-inflammatory agents (Wagner et al., 2006, Blecher et al., 2011). The anti-inflammatory activities of ZnO-NPs were assessed using several *in-vitro* assays namely COX-1, COX-2, 15-LOX, and sPLA2. The results obtained from these assays exhibited efficient inhibitory activity against all the tested concentrations of NPs. Among these, sPLA2 showed highest inhibitory potential (33.2 ± 1.71 %), followed by 15-LOX (24.3 ± 1.88 %), COX-2 (17.89 ± 1.55 %), and COX-1 (16.53 ± 1.38 %), respectively, as depicted in Fig. 5. Overall results revealed that ZnO-NPs had shown efficient inhibition of sPLA2 and 15-LOX enzymes involved in inflammatory processes.

### 3.8 ***In-vitro*** anti**-aging potential**

**Fig. 6:**
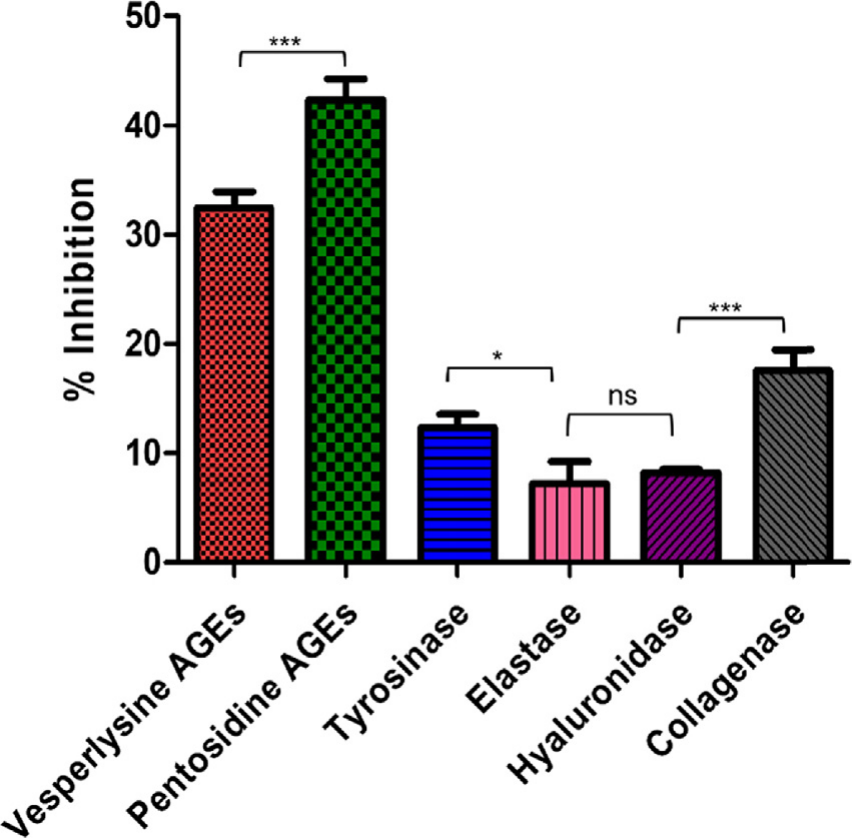
The anti-aging potential of biosynthesized ZnO-NPs.

The biosynthesized ZnO-NPs (200 μg/mL) were evaluated for their anti-aging potentials which is based on the *in-vitro* enzyme inhibition of hyaluronidase, tyrosinase, collagenase, AGEs, and elastase enzymes. Enzymes like hyaluronidase, elastase, and collagenase destroy the components of the extracellular matrix which result in the formation of deep wrinkles, loss of skin tone, and flexibility (Coricovac et al., 2015, Liyanaarachchi et al., 2018). The phenomenon of aging results in tyrosinase disorders and is considered the primary causative agent of melasma or freckles and malignant melanoma (Briganti et al., 2003). Likewise, advanced glycation end products (AGEs) are also directly connected to aging or age-related disorders due to the overproduction of oxidative species that cause oxidative stress (Gkogkolou and Böhm, 2012). Specific compounds possessing anti-aging properties are in demand for their application in cosmetic industries that can deter such enzymatic pathways or processes. Several studies reported that compounds that belong to the class III deacetylase family, such as SIRT-1, have appeared as a potent agent for survival and management of oxidative stress (Hori et al., 2013). Our current study tested biosynthesized ZnO-NPs against various enzymes to evaluate their specific inhibitory potential. Results indicated that ZnO-NPs had shown significant inhibitory activities (up to 42.32 ± 1.89 %) against pentosidine AGEs, followed by (up to 32.42 ± 1.49 %) for vesperlysine AGEs. Collagenase and tyrosinase exhibited the moderate inhibitory potential of (17.56 ± 1.88 %) and (12.3 ± 1.24 %), respectively. However, elastase and hyaluronidase have displayed the lowest inhibitory potential of 7.2 ± 2.00 and 8.19 ± 0.32, as depicted in Fig. 6. From overall findings, it has been exhibited that ZnO-NPs have efficiently inhibited two enzymes, including pentosidine AGEs and vesperlysine AGEs. ZnO is now widely declared as an anti-aging element, and its use in sunscreens and cosmetics as an active UVA/UVB-reflecting agent has been increased because it offers about 75 % protection against UVB. The use of ZnO-NPs and TiO_2_ NPs in sunscreens is further strengthened because of their ability to bind with proteins and form complexes, preventing free radicals formation and ROS production, which are involved in the aging process (Harraan 1955).

## 4 Discussion

In the current work, nanoparticles of zinc oxide were produced biologically from the dry root section of the plant *Delphinium uncinatum.* In addition to further characterizing the synthesized NPs, a number of other biological experiments were carried out. In order to determine the extent to which living molecules are involved in the creation of ZnO nanoparticles, a Fourier transform infrared spectroscopy experiment was carried out. Significant absorption spectra may be seen ranging from 500 to 4500 cm^−1^ as a consequence of these studies. The main absorption peaks for the confirmation of NPs were identified in the range at lower wave numbers. Confirmation that ZnO-NPs were produced may be seen in the peak of the ZnO stretching vibration at 755 cm^−1^. Our findings are comparable to those that have been published in the past (Abbasi et al., 2019, Jan et al., 2021, Jan et al., 2020). The XRD was carried out in order to get Zn-NPs average particle size. The XRD analysis revealed a number of different peaks. From the root portion of *D. uncinatum*, very stable crystalline NPs were identified using Debye Sharer’s equation, which had an average size of 30 nm. The diffractions peaks that were found in strong accord with the typical hexagonal wurtzite structure of ZnO NPs, in addition to being in agreement with JCPDS file no.361451. The very crystalline structure of ZnO NPs was proved by the fact that their diffraction peaks were extremely small and sharp. Previous research (Abbasi et al., 2019, Jan et al., 2021, Siripireddy and Mandal, 2017) lends credence to the findings presented here. The transmission electron microscope was used to undertake morphology-based characterization of nanoparticles. The various forms of irregular shapes and structures were observed, which confirmed the synthesis of NPs and their size range of 16–28 nm. The results of our study was also consistent with those of previously published studies (Abdo et al., 2021). After these characterizations, the zinc nanoparticles that were produced using environmentally friendly methods were examined further for a variety of antioxidant processes. ZnO-NPs were found to have the maximum radical scavenging activity when tested at a high concentration (200 µg/mL). The findings of the TAC (75.61 ± 1.06), TRP (110.34 ± 1.14 µg/mg AAE/mg), DPPH (25.12 ± 1.48 µg AAE/mg) tests demonstrated that ZnO-NPs had these characteristics. It was observed that with the increase in the concentration of ZnO-NPs, the reducing potential increased for the activities listed above. These findings are consistent with those of other studies that reported the strong antioxidant capacity of NPs derived from plants (Chandra et al., 2019, Abbasi et al., 2019). The inhibitory impact of ZnO-NPs was shown to be dose-dependent, with a noticeably greater level of inhibition being displayed at doses of 10 mg/ml and 5 mg/ml respectively. When evaluated against *Pseudomonas aeruginosa*, the antibacterial potential of NPs showed the maximum antibacterial zones at 16 0.98 mm. It has been hypothesized that the increased antibacterial activity was caused by the high surface to volume ratio (El-Belely et al 2021.,). A related study showed that a stock colloidal solution which contain a concentration of 2000 ppm of spherical ZnO-NPs that was produced by *Aspergillus terreus* strain (AF-1) having a size range of 10 to 45 nm possesses antibacterial activity against different bacterial strains. The ZnO-NPs were tested against the strains *Bacillus subtilis, Staphylococcus aureus, Escherichia coli,* and *Pseudomonas aeruginosa,* showing inhibition zones, the diameter of which ranged from 14.1 ± 0.2 to 20.2 ± 0.2 nm (Fouda et al., 2018). The NPs were tested using the in vitro –amylase inhibition assay. This essential enzyme is responsible for hydrolyzing the bonds found in polysaccharides like starch and glycogen, which ultimately results in the production of glucose and maltose. Blood glucose levels must be kept within the acceptable range in human illnesses like diabetes mellitus, therefore decreasing its activity by reversible enzymatic inhibition may be a useful tactic (Bhuyan et al 2015). This is due to the fact that one of the most crucial components of regulating blood glucose levels is ensuring that blood glucose levels stay within the acceptable range. The ZnO NPs showed a modest dose-dependent in vitro -amylase inhibition in this investigation, indicating that they could be helpful for this kind of anti-diabetes treatment. These results are supported by earlier studies conducted by (Ali et al., 2017a, Ali et al., 2017b, Jan et al., 2021). It's probable that the flavonoids and polyphenols that cover plant-based NPs are what gives them their antibacterial, antioxidant, and beta-amylase inhibitory properties (Bala et al., 2015, Rajakumar et al., 2018). ZnO-NPs were assessed for biosafety based on their non-hemolytic activity against human red blood cells. They showed a hemolytic potential of 2 percent when tested at a concentration of 10 g/mL, and this percentage gradually increased when tested at higher concentrations. Previous research also lent its support to our conclusions, as was the case here (Ali et al., 2017a, Ali et al., 2017b, Rahman et al., 2019). Multiple in-vitro assays such as COX-1, COX-2, 15-LOX, and sPLA2, were used to explore the anti-inflammatory properties of the compound. According to the findings, ZnO-NPs were able to effectively block sPLA2 (33.2 1.71 percent), which was then followed by 15-LOX (24.3 1.88 percent), these results are supported by the findings from an earlier study (Jan et al., 2021). It has been shown that ZnO-NPs have effectively inhibited two enzymes, including pentosidine AGEs and vesper lysine AGEs. These results come from the overall findings of In-vitro anti-aging potential. Our research was backed by previous work that had been published, which shown that the anti-aging potential of ZnO may be related to their capacity to absorb UV radiation and, as a result, give protection to the skin and avoid excessive damage (Jan et al., 2021).

## 5 Conclusion

This research work exhibited biosynthesis of ZnO-NPs mediated by aqueous roots extract of *Delphinium uncinatum*, a well-renowned plant for its significant medical properties. FTIR has been exploited to investigate the presence of phytochemicals or functional groups that may have participated in converting metallic zinc ions to ZnO-NPs. The purity and crystalline phases of the biosynthesized ZnO-NPs was determined by XRD analysis. TEM confirmed the almost spherical morphology of NPs. ZnO-NPs exhibited good antioxidant properties along with an intermediate inhibitory capability or anti-diabetic activity against alpha-amylase enzymes. Green synthesized ZnO-NPs have depicted an effective anti-bacterial activity. Cytotoxicity results illustrated that biosynthesized ZnO-NPs are toxic to fibroblast cells at higher concentrations, as depicted by decreased viability of NIH 3 T3 cells after exposure to ZnO-NPs.

Furthermore, ZnO-NPs exhibited comparable anti-aging potential against all tested enzymes, especially for pentosidine AGEs and vesperlysine AGEs. Likewise, as shown by the by inhibition of inflammation inducing enzymes (sPLA2 and 15-LOX), the ZnO-NPs showed efficient anti-inflammatory potential. The findings from the study concluded that biosynthesized ZnO-NPs might have the potential to be used in the cosmetics industry based on their considerable anti-aging potential and can also be used in the treatment of several disorders such as inflammatory diseases, cancer, and diabetes owing to their potent antioxidant activities. Further studies are required to evaluate, both the *in-vitro* and *in-vivo* nature of ZnO-NPs for their potential biomedical applications.

## Funding

NA.

## Declaration of Competing Interest

The authors declare that they have no known competing financial interests or personal relationships that could have appeared to influence the work reported in this paper.
